# Supplementary material for: Modeling cellular co-infection and reassortment of bluetongue virus in Culicoides midges
Source: Virus Evol. 2022 Sep 30;8(2):veac094. doi: 10.1093/ve/veac094 (PMC9662319; doi:10.1093/ve/veac094)
Supplement: veac094_Supp [file veac094_supp.zip › suppl_data/btv_culicoides_coinfection_model_supplement_20220920.pdf]

Supplementary text

The equations for the full co-infection model are as follows:

We model the infection process and viral dynamics as a system of ordinary differential equations. The equations for the **midgut** are:

$$\frac{dV_{b,i}}{dt}=-\beta_b V_{b,i}\left(T_m+E_{m,i}+E_{m,j}+E_{m,r}\right)-c_b V_{b,i}$$

$$\frac{dV_{b,j}}{dt}=-\beta_b V_{b,j}\left(T_m+E_{m,i}+E_{m,j}+E_{m,r}\right)-c_b V_{b,j}$$

$$\frac{dV_{b,r}}{dt}=-\beta_b V_{b,r}\left(T_m+E_{m,i}+E_{m,j}+E_{m,r}\right)-c_b V_{b,r}$$

$$\frac{dV_{m,i}}{dt}=-\beta_m V_{m,i}\left(T_m+E_{m,i}+E_{m,j}+E_{m,r}\right)+d_s p_s\left(I_{s,i}+I_{s,ii}\right)+\left(1-d_m\right)p_m\left(I_{m,i}+I_{m,ii}\right)-c_m V_{m,i}+\left(\frac{\omega}{2}+\frac{1-\omega}{2^{10}}\right)\left(d_s p_s\left(I_{s,ir}+I_{s,ij}\right)+\left(1-d_m\right)p_m\left(I_{m,ir}+I_{m,ij}\right)\right)$$

$$\frac{dV_{m,j}}{dt}=-\beta_m V_{m,j}\left(T_m+E_{m,i}+E_{m,j}+E_{m,r}\right)+d_s p_s\left(I_{s,j}+I_{s,jj}\right)+\left(1-d_m\right)p_m\left(I_{m,j}+I_{m,jj}\right)-c_m V_{m,j}+\left(\frac{\omega}{2}+\frac{1-\omega}{2^{10}}\right)\left(d_s p_s\left(I_{s,jr}+I_{s,ij}\right)+\left(1-d_m\right)p_m\left(I_{m,jr}+I_{m,ij}\right)\right)$$

$$\frac{dV_{m,r}}{dt}=-\beta_m V_{m,r}\left(T_m+E_{m,i}+E_{m,j}+E_{m,r}\right)+d_s p_s\left(I_{s,r}+I_{s,rr}\right)+\left(1-d_m\right)p_m\left(I_{m,r}+I_{m,rr}\right)-c_m V_{m,r}+\left(\frac{\omega}{2}+\frac{1-\omega}{2^{10}}\right)\left(d_s p_s\left(I_{s,ir}+I_{s,jr}\right)+\left(1-d_m\right)p_m\left(I_{m,ir}+I_{m,jr}\right)\right)+\left(1-\omega\right)\left(1-\right.$$ [illegible]

$$\frac{dT_m}{dt}=\mu_m T_{0,m}-\left(\beta_b\left(V_{b,i}+V_{b,j}+V_{b,r}\right)+\beta_m\left(V_{m,i}+V_{m,j}+V_{m,r}\right)+\mu_m\right)T_m$$

$$\frac{dE_{m,i}}{dt}=\left(\beta_b V_{b,i}+\beta_m V_{m,i}\right)T_m-\left(\beta_b\left(V_{b,i}+V_{b,j}+V_{b,r}\right)+\beta_m\left(V_{m,i}+V_{m,j}+V_{m,r}\right)+\frac{1}{\epsilon_m}+\mu_m\right)E_{m,i}$$

$$\frac{dE_{m,j}}{dt}=\left(\beta_b V_{b,j}+\beta_m V_{m,j}\right)T_m-\left(\beta_b\left(V_{b,i}+V_{b,j}+V_{b,r}\right)+\beta_m\left(V_{m,i}+V_{m,j}+V_{m,r}\right)+\frac{1}{\epsilon_m}+\mu_m\right)E_{m,j}$$

$$\frac{dE_{m,r}}{dt}=\left(\beta_b V_{b,r}+\beta_m V_{m,r}\right)T_m-\left(\beta_b\left(V_{b,i}+V_{b,j}+V_{b,r}\right)+\beta_m\left(V_{m,i}+V_{m,j}+V_{m,r}\right)+\frac{1}{\epsilon_m}+\mu_m\right)E_{m,r}$$

$$\frac{dE_{m,ii}}{dt}=\left(\beta_b V_{b,i}+\beta_m V_{m,i}\right)E_{m,i}-\left(\frac{1}{\epsilon_m}+\mu_m\right)E_{m,ii}$$

$$\frac{dE_{m,jj}}{dt}=\left(\beta_b V_{b,j}+\beta_m V_{m,j}\right)E_{m,i}-\left(\frac{1}{\epsilon_m}+\mu_m\right)E_{m,jj}$$

$$\frac{dE_{m,rr}}{dt}=\left(\beta_b V_{b,r}+\beta_m V_{m,r}\right)E_{m,r}-\left(\frac{1}{\epsilon_m}+\mu_m\right)E_{m,rr}$$

$$\frac{dE_{m,ij}}{dt}=\left(\beta_b V_{b,i}+\beta_m V_{m,i}\right)E_{m,j}+\left(\beta_b V_{b,j}+\beta_m V_{m,j}\right)E_{m,i}-\left(\frac{1}{\epsilon_m}+\mu_m\right)E_{m,ij}$$

$$\frac{dE_{m,ir}}{dt}=\left(\beta_b V_{b,i}+\beta_m V_{m,i}\right)E_{m,r}+\left(\beta_b V_{b,r}+\beta_m V_{m,r}\right)E_{m,i}-\left(\frac{1}{\epsilon_m}+\mu_m\right)E_{m,ir}$$

$$\frac{dE_{m,jr}}{dt}=\left(\beta_b V_{b,j}+\beta_m V_{m,j}\right)E_{m,r}+\left(\beta_b V_{b,r}+\beta_m V_{m,r}\right)E_{m,j}-\left(\frac{1}{\epsilon_m}+\mu_m\right)E_{m,jr}$$

$$\frac{dI_{m,i}}{dt}=\frac{E_{m,i}}{\epsilon_m}-\mu_m^{(i)}I_{m,i}$$

$$\frac{dI_{m,j}}{dt}=\frac{E_{m,j}}{\epsilon_m}-\mu_m^{(j)}I_{m,j}$$

$$\frac{dI_{m,r}}{dt}=\frac{E_{m,r}}{\epsilon_m}-\mu_m^{(r)}I_{m,r}$$

$$\frac{dI_{m,ii}}{dt} = \frac{E_{m,ii}}{\epsilon_m} - \mu_m^{(i)} I_{m,ii}$$

$$\frac{dI_{m,jj}}{dt} = \frac{E_{m,jj}}{\epsilon_m} - \mu_m^{(i)} I_{m,jj}$$

$$\frac{dI_{m,rr}}{dt} = \frac{E_{m,rr}}{\epsilon_m} - \mu_m^{(i)} I_{m,rr}$$

$$\frac{dI_{m,ij}}{dt} = \frac{E_{m,ij}}{\epsilon_m} - \mu_m^{(i)} I_{m,ij}$$

$$\frac{dI_{m,ir}}{dt} = \frac{E_{m,ir}}{\epsilon_m} - \mu_m^{(i)} I_{m,ir}$$

$$\frac{dI_{m,jr}}{dt} = \frac{E_{m,jr}}{\epsilon_m} - \mu_m^{(i)} I_{m,jr}$$

The equations for the **secondary tissues** are:

$$\frac{dV_{s,i}}{dt} = -\beta_s V_{s,i}\left(T_s + E_{s,i} + E_{s,j} + E_{s,r}\right) + d_m p_m\left(I_{m,i} + I_{m,ii}\right) + \left(1-d_s\right) p_s\left(I_{s,i} + I_{s,ii}\right) - c_s V_{s,i} + \left(\frac{\omega}{2} + \frac{1-\omega}{2^{10}}\right)\left(d_m p_m\left(I_{m,ir} + I_{m,ij}\right) + \left(1-d_s\right) p_s\left(I_{m,ir} + I_{s,ij}\right)\right)$$

$$\frac{dV_{s,j}}{dt} = -\beta_s V_{s,j}\left(T_s + E_{s,i} + E_{s,j} + E_{s,r}\right) + d_m p_m\left(I_{m,j} + I_{m,jj}\right) + \left(1-d_s\right) p_s\left(I_{m,j} + I_{s,jj}\right) - c_s V_{s,j} + \left(\frac{\omega}{2} + \frac{1-\omega}{2^{10}}\right)\left(d_m p_m\left(I_{m,jr} + I_{m,ij}\right) + \left(1-d_s\right) p_s\left(I_{s,jr} + I_{s,ij}\right)\right)$$

$$\frac{dV_{s,r}}{dt} = -\beta_s V_{s,r}\left(T_s + E_{s,i} + E_{s,j} + E_{s,r}\right) + d_m p_m\left(I_{m,r} + I_{m,rr}\right) + \left(1-d_s\right) p_s\left(I_{m,r} + I_{s,rr}\right) - c_s V_{s,r} + \left(\frac{\omega}{2} + \frac{1-\omega}{2^{10}}\right)\left(d_m p_m\left(I_{m,ir} + I_{m,jr}\right) + \left(1-d_s\right) p_s\left(I_{s,ir} + I_{s,jr}\right)\right) + \left(1-\omega\right)\left(1 - \frac{1}{2^9}\right. \text{[illegible]}$$

$$\frac{dT_s}{dt} = \mu_s T_{0,s} - \left(\beta_s\left(V_{s,i} + V_{s,j} + V_{s,r}\right) + \mu_s\right) T_s$$

$$\frac{dE_{s,i}}{dt}=\beta_s V_{s,i} T_s-\left(\beta_s\left(V_{s,i}+V_{s,j}+V_{s,r}\right)+\frac{1}{\epsilon_s}+\mu_s\right)E_{s,i}$$

$$\frac{dE_{s,j}}{dt}=\beta_s V_{s,j} T_s-\left(\beta_s\left(V_{s,i}+V_{s,j}+V_{s,r}\right)+\frac{1}{\epsilon_s}+\mu_s\right)E_{s,j}$$

$$\frac{dE_{s,r}}{dt}=\beta_s V_{s,r} T_s-\left(\beta_s\left(V_{s,i}+V_{s,j}+V_{s,r}\right)+\frac{1}{\epsilon_s}+\mu_s\right)E_{s,r}$$

$$\frac{dE_{s,ii}}{dt}=\beta_s V_{s,i} E_{s,i}-\left(\frac{1}{\epsilon_s}+\mu_s\right)E_{s,ii}$$

$$\frac{dE_{s,jj}}{dt}=\beta_s V_{s,j} E_{s,i}-\left(\frac{1}{\epsilon_s}+\mu_s\right)E_{s,jj}$$

$$\frac{dE_{s,rr}}{dt}=\beta_s V_{s,r} E_{s,r}-\left(\frac{1}{\epsilon_s}+\mu_s\right)E_{s,rr}$$

$$\frac{dE_{s,ij}}{dt}=\beta_s V_{s,i} E_{s,j}+\beta_s V_{s,j} E_{s,i}-\left(\frac{1}{\epsilon_s}+\mu_s\right)E_{s,ij}$$

$$\frac{dE_{s,ir}}{dt}=\beta_s V_{s,i} E_{s,r}+\beta_s V_{s,r} E_{s,i}-\left(\frac{1}{\epsilon_s}+\mu_s\right)E_{s,ir}$$

$$\frac{dE_{s,jr}}{dt}=\beta_s V_{s,j} E_{s,r}+\beta_s V_{s,r} E_{s,j}-\left(\frac{1}{\epsilon_s}+\mu_s\right)E_{s,jr}$$

$$\frac{dI_{s,i}}{dt}=\frac{E_{s,i}}{\epsilon_s}-\mu_s^{(i)} I_{s,i}$$

$$\frac{dI_{s,j}}{dt} = \frac{E_{s,j}}{\epsilon_s} - \mu_s^{(j)} I_{s,j}$$

$$\frac{dI_{s,r}}{dt} = \frac{E_{s,r}}{\epsilon_s} - \mu_s^{(r)} I_{s,r}$$

$$\frac{dI_{s,ii}}{dt} = \frac{E_{s,ii}}{\epsilon_s} - \mu_s^{(i)} I_{s,ii}$$

$$\frac{dI_{s,jj}}{dt} = \frac{E_{s,jj}}{\epsilon_s} - \mu_s^{(i)} I_{s,jj}$$

$$\frac{dI_{s,rr}}{dt} = \frac{E_{s,rr}}{\epsilon_s} - \mu_s^{(i)} I_{s,rr}$$

$$\frac{dI_{s,ij}}{dt} = \frac{E_{s,ij}}{\epsilon_s} - \mu_s^{(i)} I_{s,ij}$$

$$\frac{dI_{s,ir}}{dt} = \frac{E_{s,ir}}{\epsilon_s} - \mu_s^{(i)} I_{s,ir}$$

$$\frac{dI_{s,jr}}{dt} = \frac{E_{s,jr}}{\epsilon_s} - \mu_s^{(i)} I_{s,jr}$$

Supplementary tables

Table S1: Model parameters included in calibration and sensitivity analysis, the ranges explored (i.e. limits of the uniform priors for the calibration), and calibrated values (i.e. *maximum a posteriori* values and 95% credible interval)

Symbol	Definition	Units	Explored range	Calibrated values (MAP; 95% CI)
$\beta_m T_{0,m}$	The rate at which virions enter uninfected target cells	$hour^{-1}$	$[5x10^{-4}, 5]$	1.90; [1.88, 2.04]
d_m	The proportion of virions produced in the midgut which pass the MEB	-	[0, 1]	0.385; [0.385, 0.495]
p_m	The rate at which productively infected midgut cells produce new virions	virions $cell^{-1}$ $hour^{-1}$	$[1, 10^4]$	194; [194, 225]
$T_{0,m}$	The initial number of target cells in the midgut	cells	$[1, 10^4]$	4010; [3830, 4030]
μ_m	The midgut target cell death rate	$hour^{-1}$	[0.01, 1]	1.00; [0.975, 1.00]
ε_m	The average length of the eclipse phase in midgut cells	hours	[0.0167, 120]	116; [111, 116]
c_s	The viral death rate in the secondary tissues	$hour^{-1}$	[0.01, 1]	0.448; [0.432, 0.590]
β_s	The per-virion rate of effective contact between cells and virions in the secondary tissues	$hour^{-1}$ $cell^{-1}$	$[10^{-7}, 10^{-5}]$	$8.63x10^{-7}$; $[0.817, 1.40]x10^{-6}$
d_s	The proportion of virions produced in the secondary tissues which return to the midgut	-	[0.01, 0.99]	0.138; [0.129,0.141]
p_s	The rate at which	virions $cell^{-1}$ $hour^{-1}$	$[1, 10^4]$	9990;

	productively infected secondary tissue cells produce new virions			[5160, 9980]
$T_{0,s}$	The initial number of target cells in the secondary tissues	cells	[1, 10^4]	5360;<br>[4930, 5390]
μ_s	The secondary tissue target cell death rate	hour^{-1}	[0.01, 1]	0.996;<br>[0.751, 0.999]
ε_s	The average length of the eclipse phase in secondary tissue cells	hours	[0.0167, 120]	96.6;<br>[95.7, 101]
ω	The proportion of reassortants produced by co-infected cells that are withlike	-	[0, 1]	0.54;<br>[0.504, 0.626]
p_{DB}^{inc}	The increase on the logistic scale in the probability of the dissemination barrier being crossed following a second bloodmeal	logistic(virions cell^{-1} hour^{-1})	[0, 10]	9.38;<br>[9.31, 9.53]

Supplementary figures

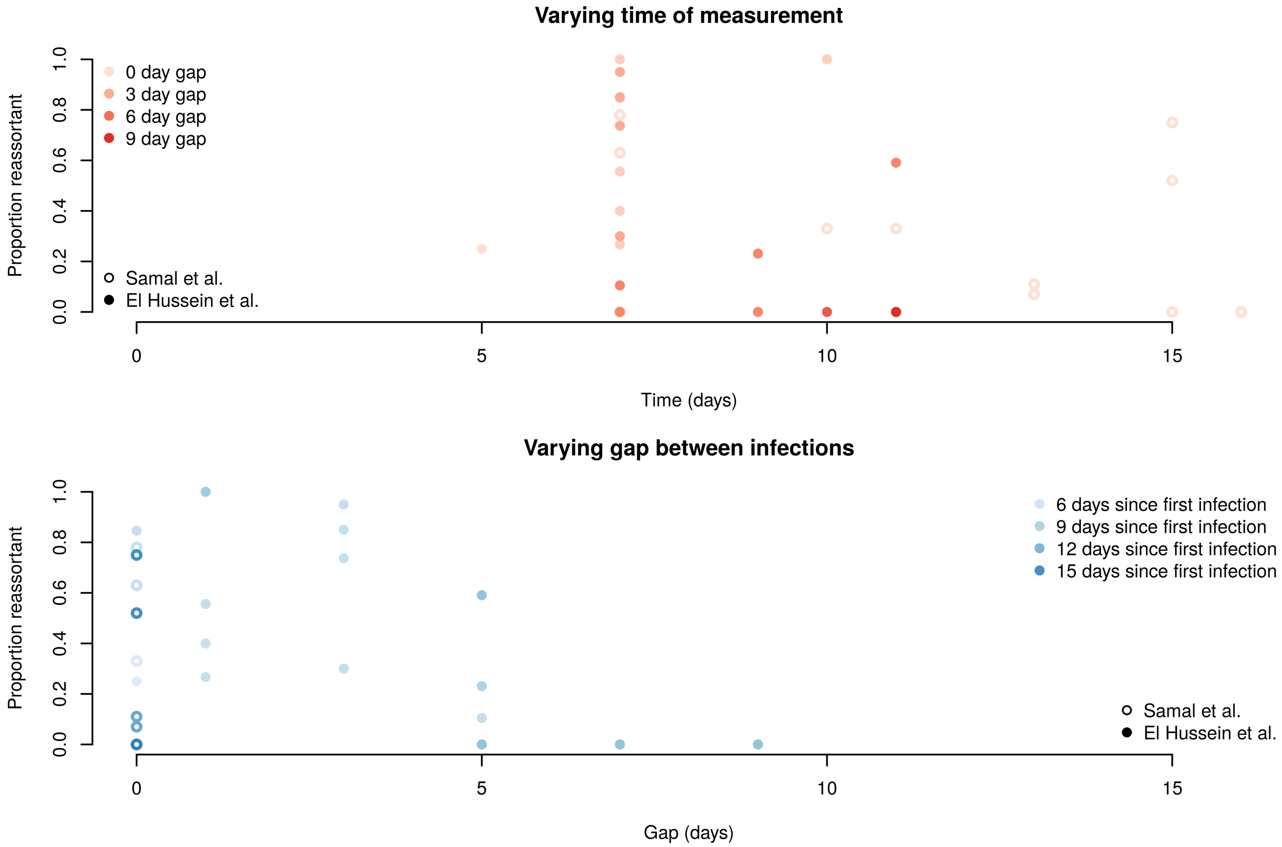

Fig S1: Data on proportion of reassortant virions from el Hussein et al. and Samal et al. [REFs]

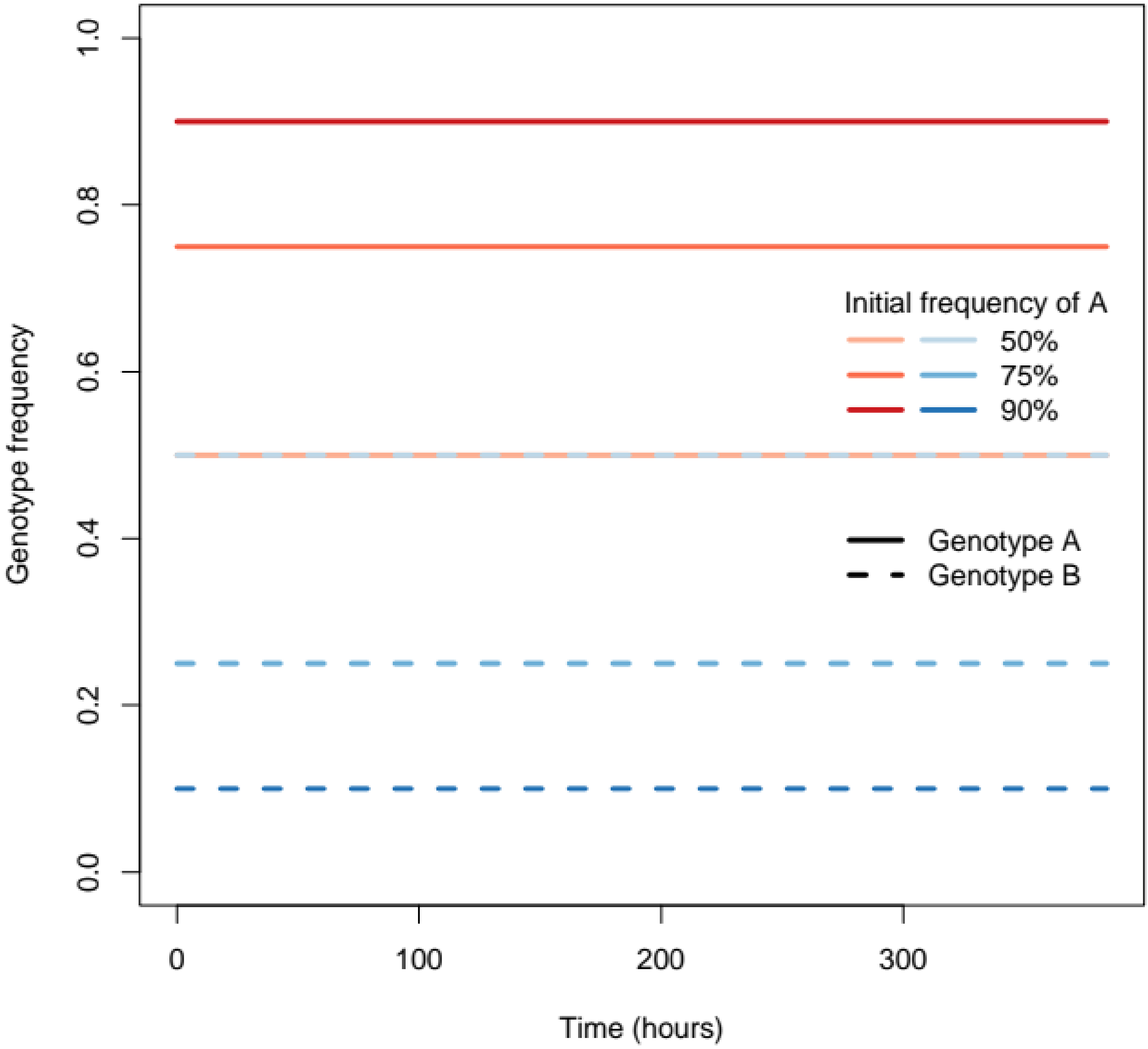

Fig. S2. If both genotypes have the same parameters, then each exhibits stable frequencies under coinfection without reassortment. I.e., there is no a priori stable strain frequency built into the model.

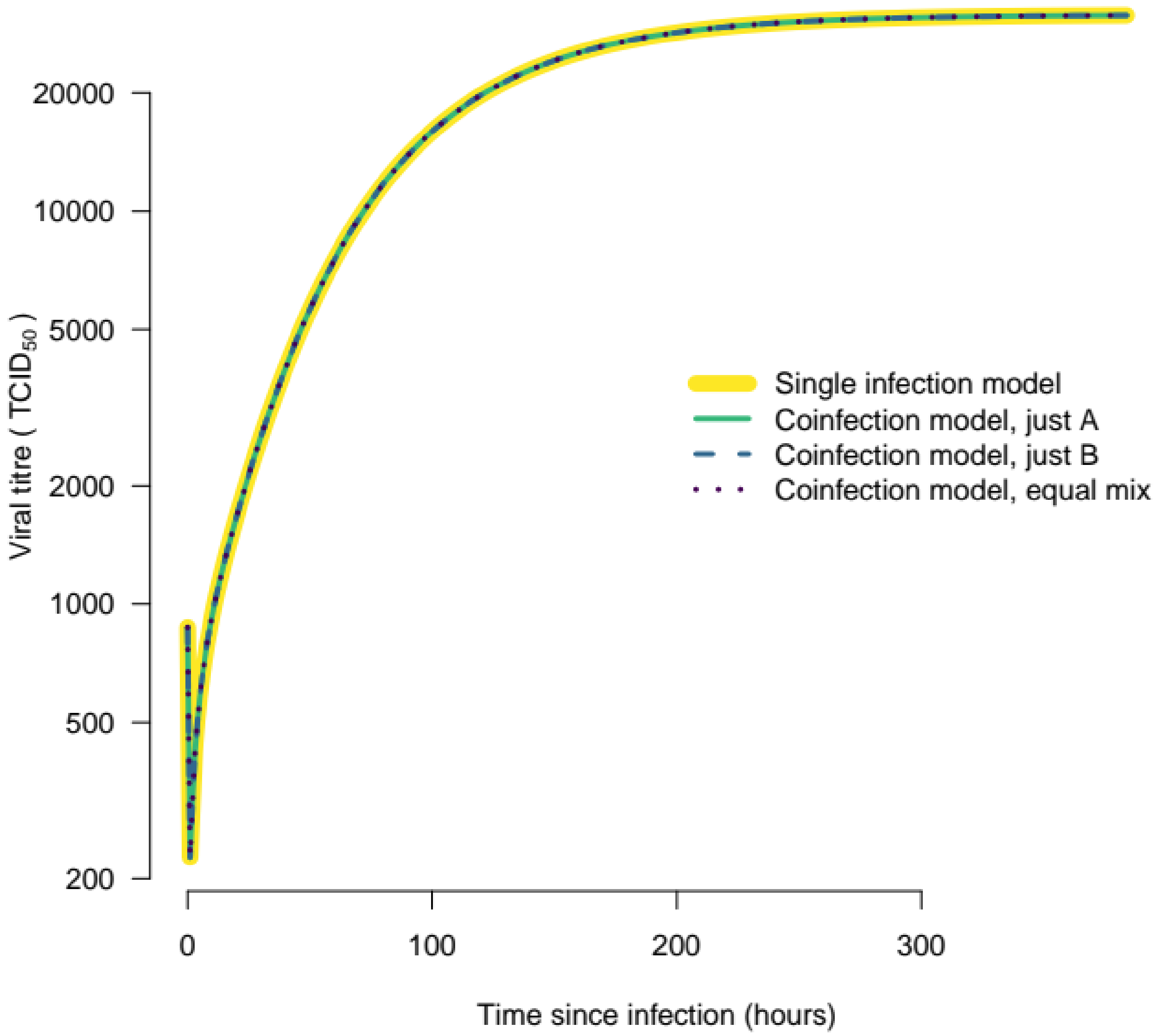

Fig. S3: In the absence of reassortment, the single model and the coinfection model behave identically, irrespective of the initial mix of virus types in the bloodmeal (and assuming both types have the same parameters)

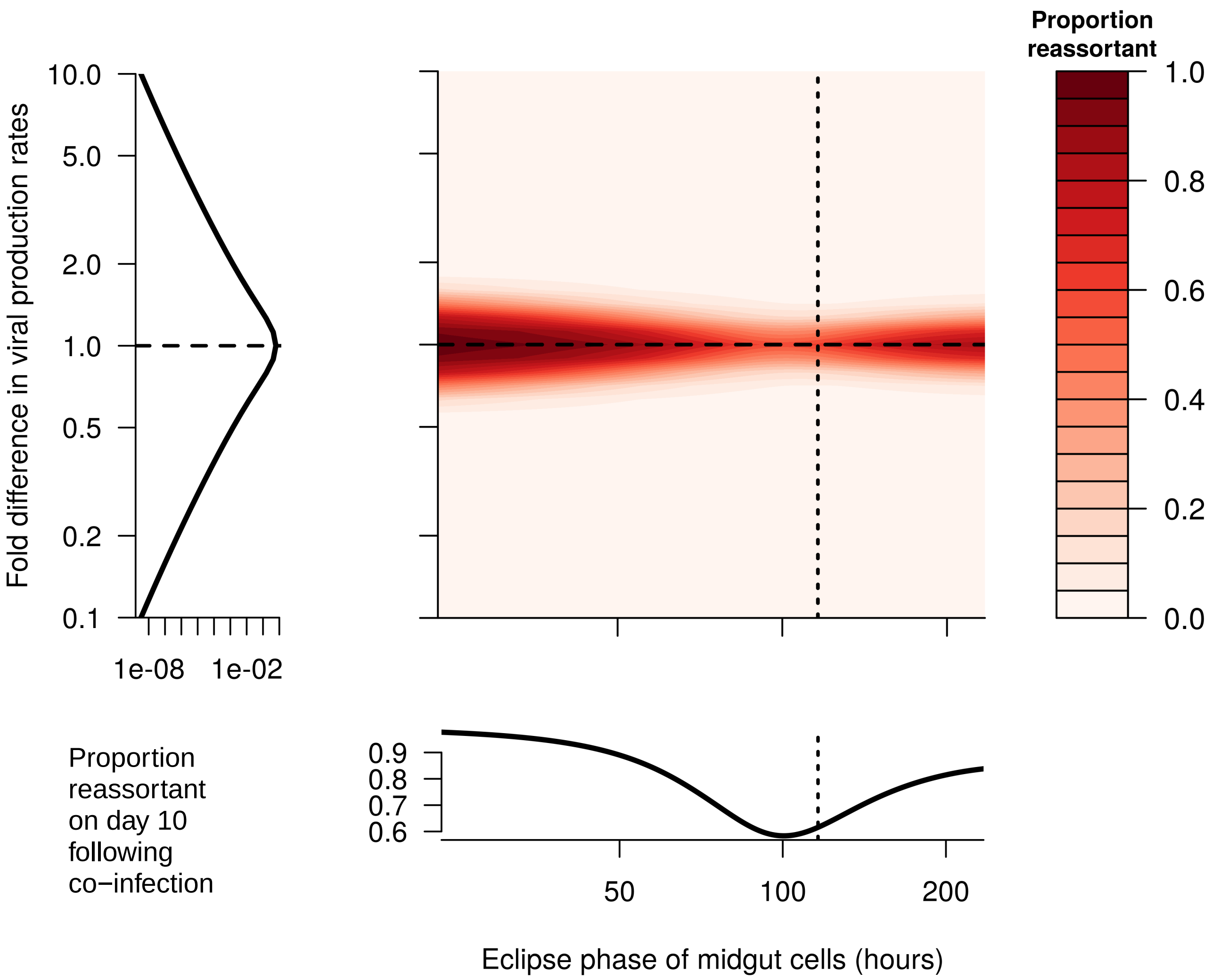

Fig. S4: A: The proportion of reassortant viruses observed ten days after the initial infection (color-scale) when the blood meals occur on day 0, for different lengths of eclipse phase (x-axis) and for different viral production rates between the two infections (y-axis). When the fold-change in p is less than 1, the first infecting virus has higher production rate than the second, and when it is greater 1 the second virus has higher production rate. Dashed lines represent baseline values. Note that the eclipse phase in the secondary tissues was also varied by proportionally the same amount from baseline as the eclipse phase in the midgut (not shown).

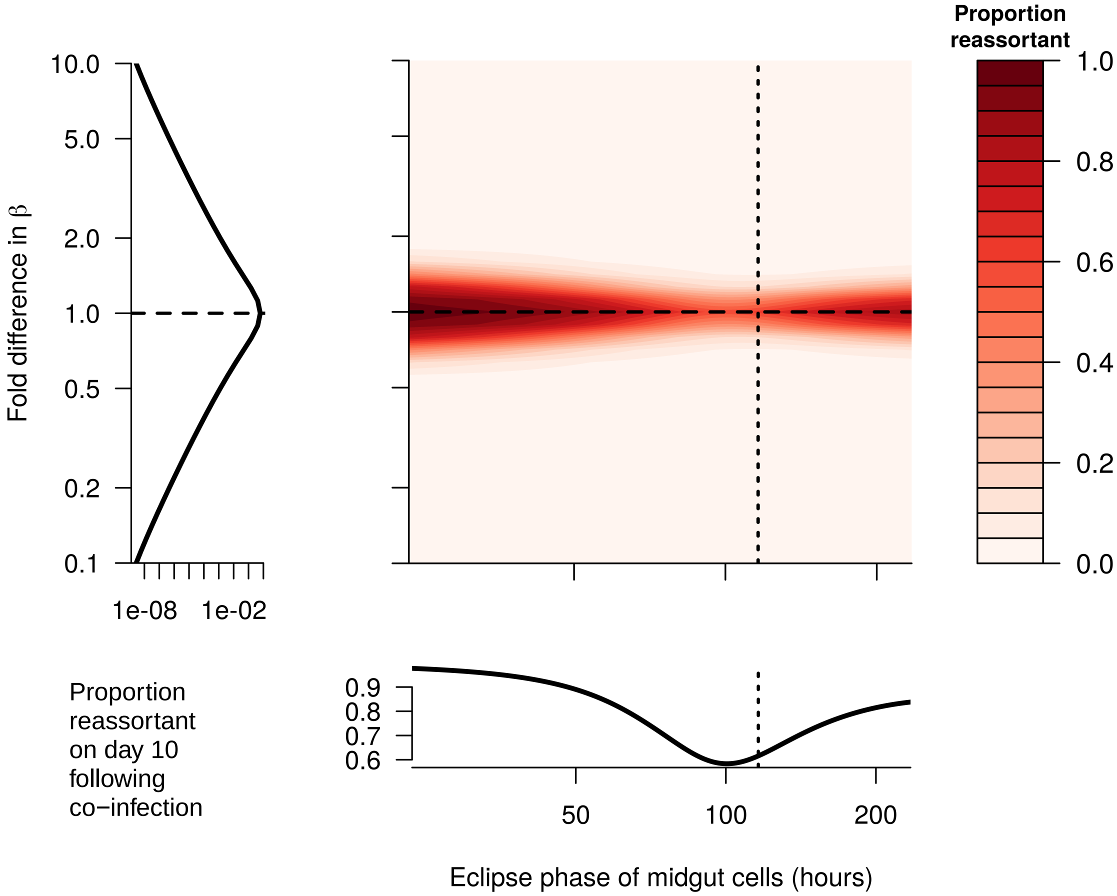

Fig. S5: A: The proportion of reassortant viruses observed ten days after the initial infection (color-scale) when the blood meals occur on day 0, for different lengths of eclipse phase (x-axis) and for different rates of effective contact between cells and viruses (β) between the two infections (y-axis). When the fold-change in β is less than 1, the first infecting virus has higher rate of effective contact than the second, and when it is greater 1 the second virus has higher rate of effective contact. Dashed lines represent baseline values. Note that the eclipse phase in the secondary tissues was also varied by proportionally the same amount from baseline as the eclipse phase in the midgut (not shown).

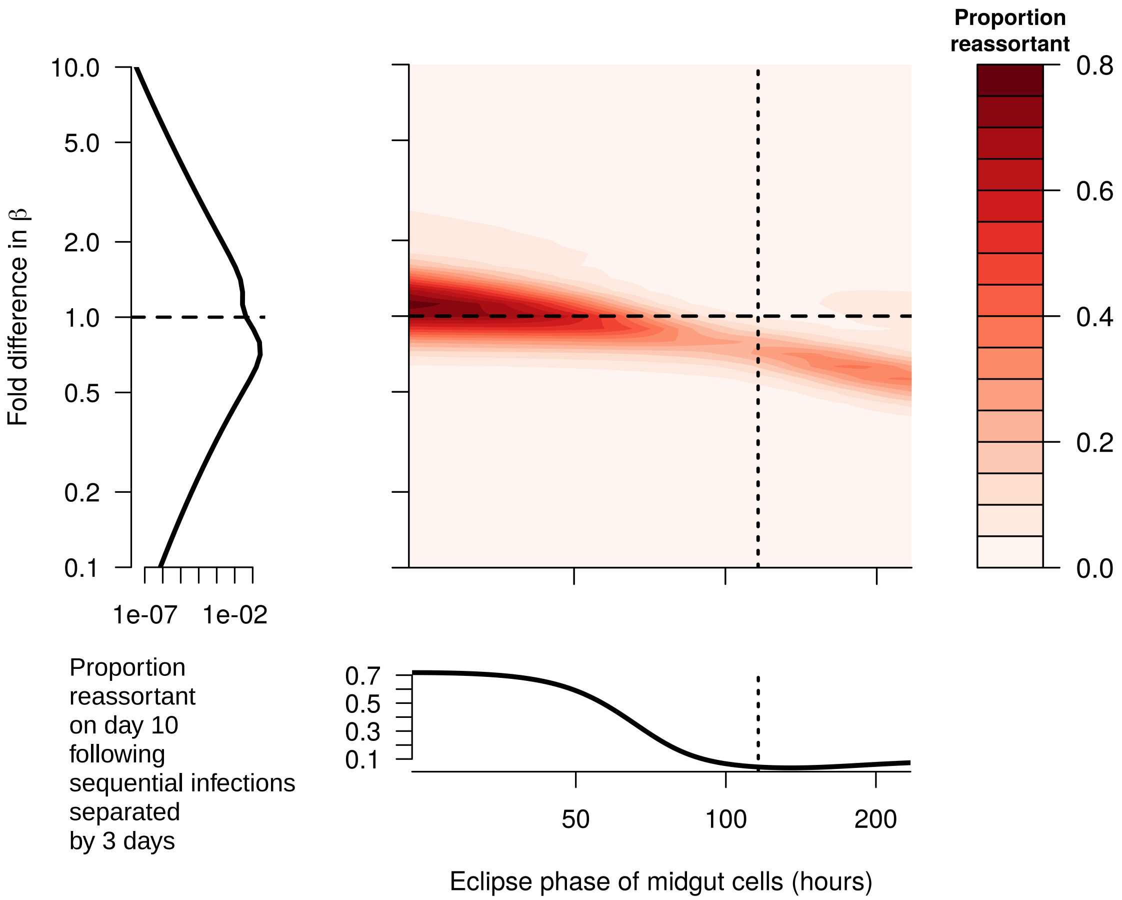

Fig. S6: The proportion of reassortant viruses observed ten days after the initial infection (color-scale) when the second bloodmeal occurs three days after the initial infection, for different lengths of eclipse phase (x-axis) and for different rates of effective contact between the two infections (y-axis). When the fold-change in β is less than 1, the first infecting virus has higher effective contact rate than the second, and when it is greater than 1 the second virus has higher effective contact rate. Dashed lines represent baseline values. Note that the eclipse phase in the secondary tissues was also varied by proportionally the same amount from baseline as the eclipse phase in the midgut (not shown). Note that the left hand plot has an x-axis that is on a log-scale – reassortment decreases very quickly as the difference in viral fitness increases.

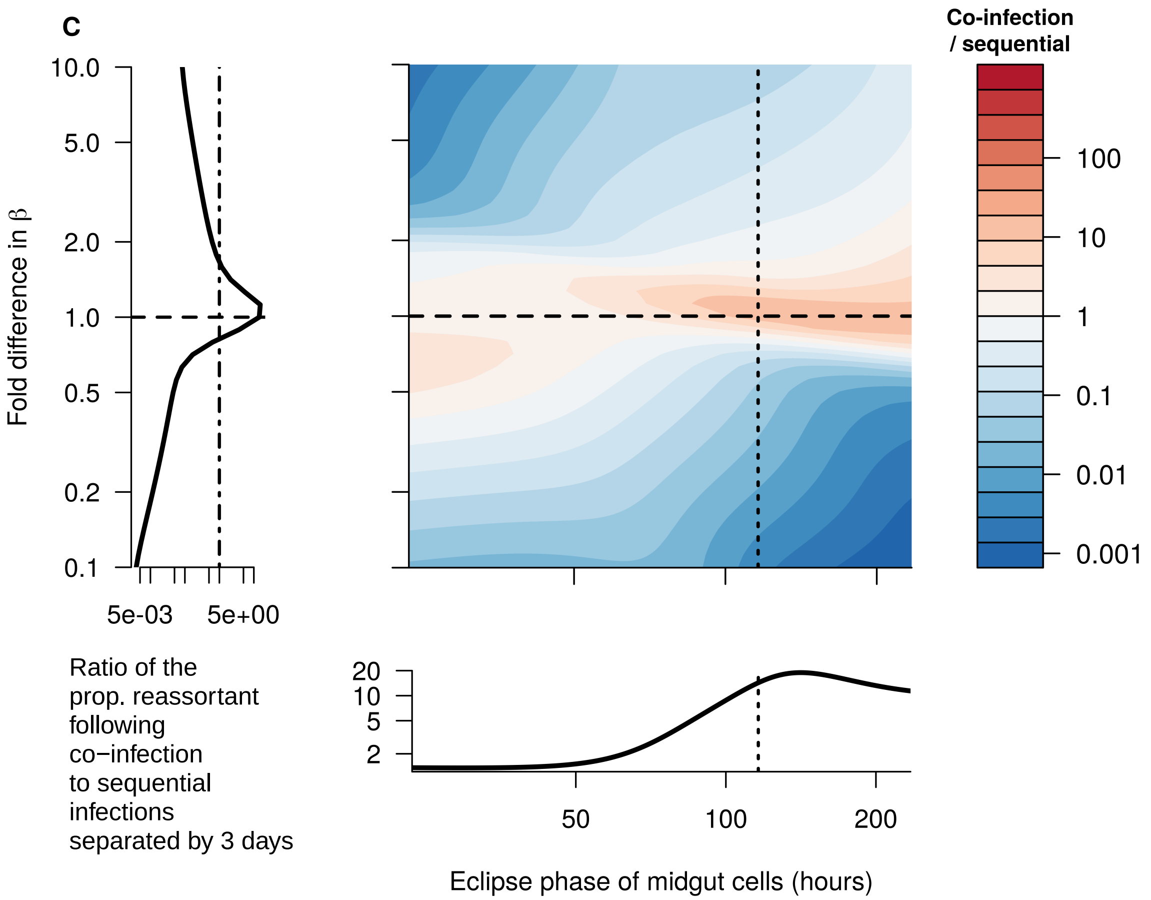

Fig. S7: The ratio of the proportion of reassortant viruses observed 10 days after the initial infection when both bloodmeals occur on day zero to the same proportion when the second bloodmeal occurs on day three (color-scale), for different lengths of eclipse phase (x-axis) and for different rate of effective contact between the two infections (y-axis). When this ratio is below 1, it indicates an increase in the proportion of viruses that are reassortant, as observed in el Hussein et al. When the fold-change in β is less than 1, the first infecting virus has higher effective contact rate than the second, and when it is greater than 1 the second virus has higher effective contact rate. Dashed lines represent baseline values. Note that the eclipse phase in the secondary tissues was also varied by proportionally the same amount from baseline as the eclipse phase in the midgut (not shown). Note also that the left hand plot has an x-axis that is on a log-scale.

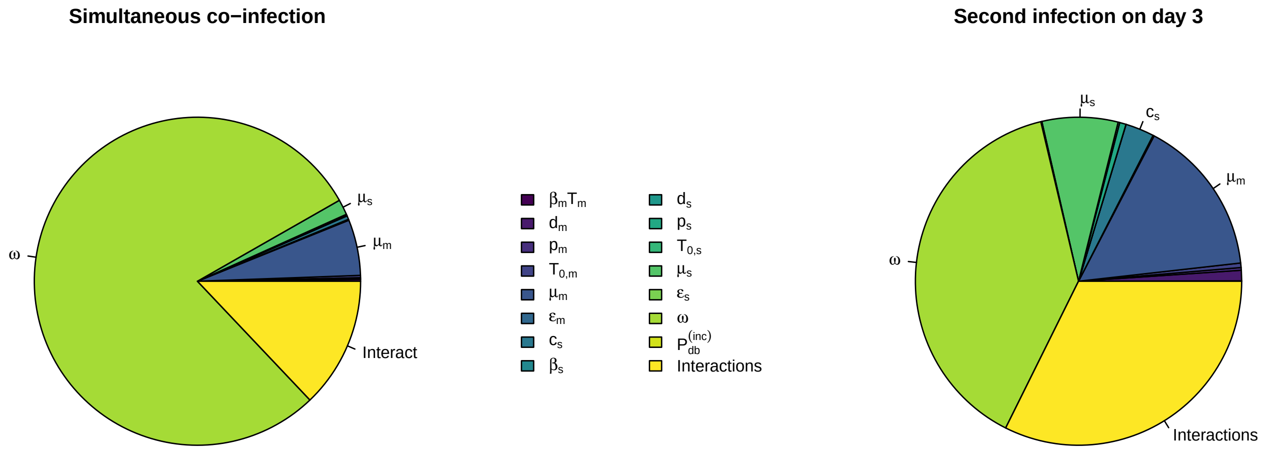

Fig. S8: First-order indices for Saltelli variance-based sensitivity analysis of the proportion of viruses that are reassortant. Pie segments represent the variance explained by each parameter on its own, with the final segment describing the amount of variance explained by interactions between parameters. The most important parameters are labelled: ω is the proportion of virions produced by a coinfected cell that are with-like (i.e. are not reassortants), μ is the rate of cellular death, and c is the rate of viral clearance/death. Left panel shows the indices for the proportion reassortant on day 10 following a simultaneous coinfection on day 0. Right panel is the same, but for infection on day 3.

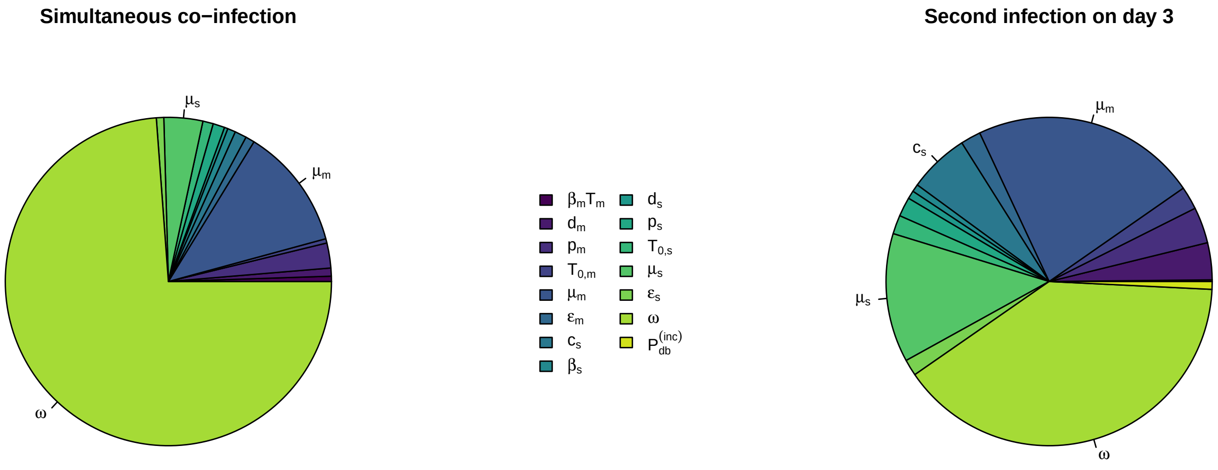

Fig. S9: Total-order indices for Saltelli variance-based sensitivity analysis of the proportion of viruses that are reassortant. Pie segments represent the variance explained by each parameter, both on its own and in interactions with all other parameters. The most important parameters are labelled: ω is the proportion of virions produced by a coinfected cell that are with-like (i.e. are not reassortants), μ is the rate of cellular death, and c is the rate of viral clearance/death. Left panel shows the indices for the proportion reassortant on day 10 following a simultaneous coinfection on day 0. Right panel is the same, but for infection on day 3.
